# Clinical and Radiographic Assessment of Milled Versus 3D-Printed Patient-Specific Subperiosteal Implants for Atrophic Mandibular Ridges: A Randomized Clinical Trial

**DOI:** 10.7759/cureus.80326

**Published:** 2025-03-10

**Authors:** Saad A Darwish, Wael A El-Mohandes, Bahaa El-Din Abd Rabbo

**Affiliations:** 1 Department of Oral and Maxillofacial Surgery, Faculty of Dental Medicine, Al-Ahzar University, Cairo, EGY

**Keywords:** 3d printing, atrophic mandible, cad/cam milling, digital planning, subperiosteal implants

## Abstract

Objective: This study evaluated the clinical and radiographic outcomes of patient-specific subperiosteal implants fabricated via milling or 3D printing for mandibular rehabilitation in patients with severely atrophic mandibles.

Methodology: Twenty patients with severely atrophic edentulous mandibular ridges were randomly assigned to receive subperiosteal implants manufactured by Computer-Aided Design/Computer-Aided Manufacturing (CAD/CAM) milling or 3D printing. Preoperative cone beam computed tomography scans and digital planning generated customized stereolithography (STL) files that guided the fabrication of implants from titanium grade 4 using either subtractive (milling) or additive (3D printing) techniques. After creating a full-thickness mucoperiosteal flap, the custom-made implants were seated and secured with mini-screws, and patients received written postoperative care instructions and medications. Primary outcomes included implant survival, bone resorption, and placement accuracy assessed by measuring mesiodistal and buccolingual angular deviations. Secondary outcomes comprised the duration of the intervention and soft tissue dehiscence evaluated at multiple postoperative intervals up to 12 months. Data were analyzed using the chi-square test for categorical data and the t-test for continuous data.

Results: There were no statistically significant differences between the two groups regarding implant survival, with one implant failing in the milling group (*P* = 0.869). Changes in bone resorption between the baseline and after 12 months showed no statistically significant results between the two groups (*P* = 0.1396). The angular deviation between the planned abutments and the postoperative scans showed no statistically significant results between the two groups regarding the mesiodistal and bucco-lingual deviations (*P* > 0.05). The two groups showed similar results in soft tissue dehiscence and duration of the treatment, with no significant differences between them (*P* > 0.05).

Conclusions: Both milling and 3D printing techniques offer effective and comparable solutions for fabricating patient-specific subperiosteal implants to rehabilitate atrophic mandibles. Available resources and clinician expertise should inform the choice between these methods.

## Introduction

Missing teeth can negatively affect social confidence and self-esteem by compromising oral function and aesthetics. As life expectancy rises and the quality of life improves, the need for effective dental rehabilitation solutions has grown [1]. The treatment of atrophic mandibles presents a significant challenge in implant dentistry, requiring careful planning and innovative solutions [2]. Traditional treatment options, such as short implants, nerve lateralization, and bone grafting before implant placement, have significant drawbacks, including prolonged treatment duration, higher costs, possible complications, and patient-related limitations stemming from medical status or personal preferences [3,4].

The concept of cortically fixed implants has emerged as an alternative. These implants use cortical buttresses to improve retention and stability, allowing for a single-stage surgical approach. This marks a significant advancement over Linkow’s subperiosteal implant technique, which necessitated two separate surgeries. The initial surgery involved taking physical impressions with silicone, followed by a second surgery to place the framework, which relied solely on mucoperiosteal retention [5,6]. However, cortically fixed implants may not be suitable for all patients, particularly those with extensive atrophy with minimal bone engagement.

Advancements in digital planning have enabled the development of patient-specific subperiosteal implants (PSI) tailored to individual anatomical structures using osteosynthesis screw fixation [7,8]. Integrating computed tomography (CT) scans with specialized software enables precise diagnosis, efficient treatment planning, and the development of customized stereolithography files (STL). These digital models simplify the fabrication of PSIs, providing better fit, stability, and functional outcomes [9-13].

Two primary manufacturing methods for PSIs are milling and 3D printing. Milling, a subtractive technique, carves the implant from a solid block, while 3D printing, an additive process, constructs the implant layer by layer from a digital design [9-11,13]. These manufacturing techniques enable the production of precise surgical guides and custom prosthetics, reducing operating room time, enhancing surgical accuracy, and minimizing trauma to vital structures. Additionally, CT-guided procedures improve implant placement precision, reducing tissue manipulation and promoting faster recovery [14,15].

In light of these advancements, this study aims to evaluate PSIs' clinical and radiographic outcomes in rehabilitating atrophic mandibles and comparing the effectiveness of milling and 3D printing techniques regarding implant survival, accuracy, bone resorption, soft tissue dehiscence, and duration of the intervention.

## Materials and methods

Trial design

This study was designed as a randomized parallel-arm clinical trial with a 1:1 allocation ratio and a superiority framework. The research ethics committee of the Faculty of Dental Medicine, Al-Azhar University, approved the study protocol with the ethical code 676/249. The study was reported according to the Consolidated Standards of Reporting Trials (CONSORT) guidelines for reporting randomized clinical trials [16]. The protocol was retrospectively registered at Open Science Framework with the following ID: https://doi.org/10.17605/OSF.IO/7DEV3.

Participants and setting

This study included 20 patients with atrophic mandibular alveolar ridges seeking fixed implant-supported prostheses. The patients were selected from those attending the outpatient clinic of the Oral and Maxillofacial Surgery Department, Faculty of Dental Medicine, Al-Azhar University, Cairo, Egypt.

Eligible participants had severely atrophic edentulous mandibles characterized by a ridge height less than 6 mm above the inferior alveolar nerve (IAN), including cases following mandibular marginal resection. Participants in the trial were excluded if they had uncontrolled systemic diseases affecting bone healing, any pathology at the surgical site, were heavy smokers (defined as smoking more than 20 cigarettes daily) or had received radiotherapy to the head and neck area in the past 12 months due to current or prior malignancies.

Interventions

All patients received a comprehensive preoperative evaluation, including clinical and radiographic assessments, a complete dental history, and a detailed medical history to identify systemic conditions. Cone beam computed tomography (CBCT) scans were performed using the Planmeca Promax 3D Classic (Planmeca, Helsinki, Finland), with an 11 cm × 8 cm field of view at 90 kV (pulsed mode) and 12.5 mA.

Small gutta-percha markers were applied to the patient's non-radiopaque denture constructed preoperatively. Scans were obtained with and without the denture. The DICOM (Digital Imaging and Communications in Medicine) header was inspected for movement artifacts or air pockets; if the implant area was unaffected, image segmentation proceeded, with motion artifacts further assessed via 3D volume rendering.

**Figure 1:**
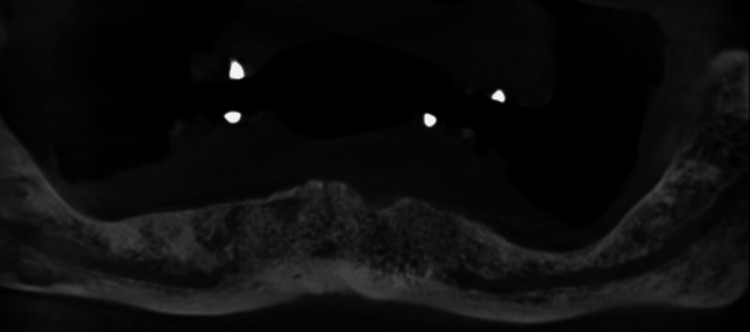
Panoramic reconstruction view obtained from preoperative CBCT. CBCT, cone beam computed tomography

**Figure 2:**
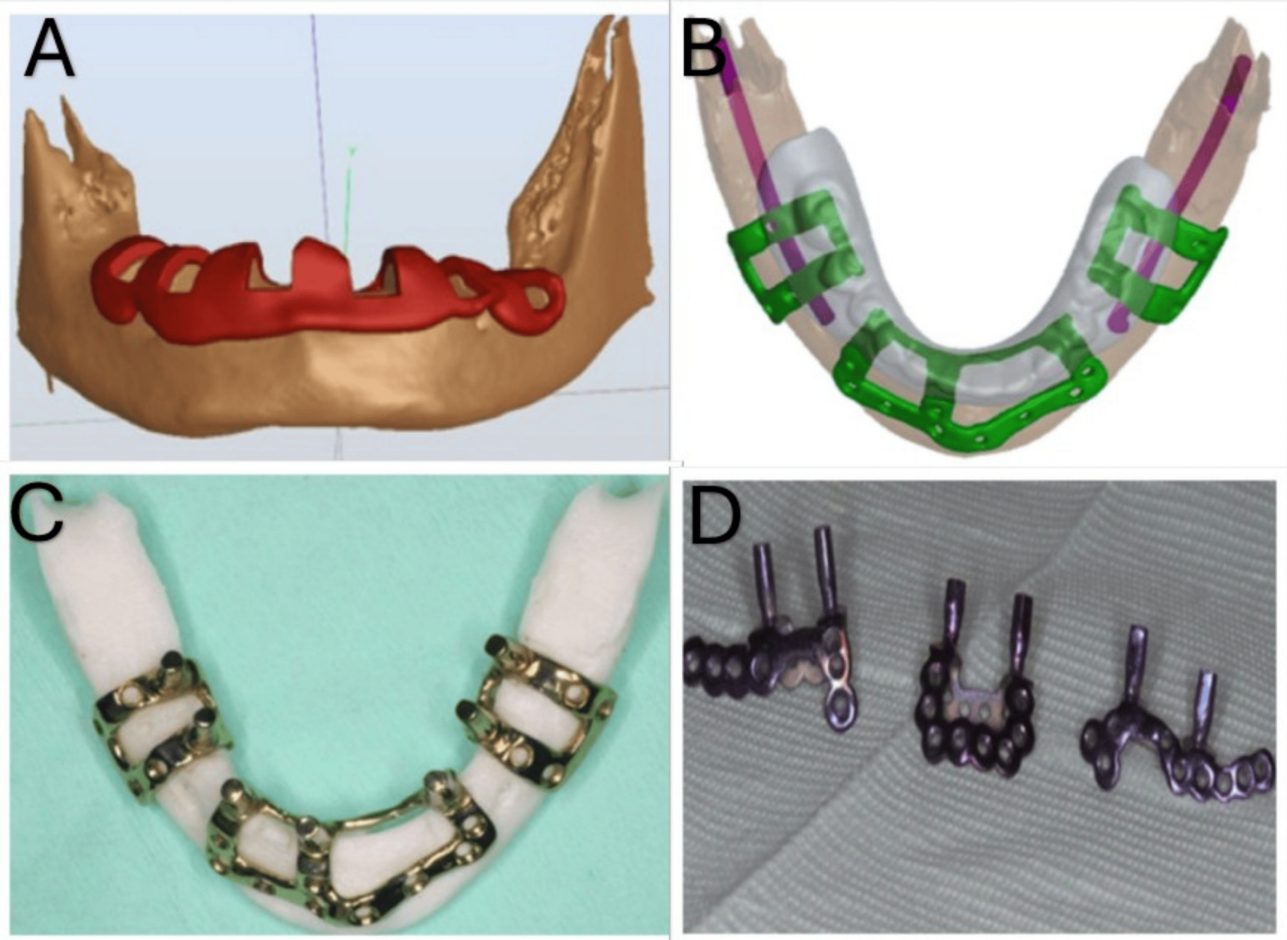
Virtual planning and manufacturing of the subperiosteal implant. (A) Virtual cutting guide for the grooves. (B) Virtual prosthetic-driven plan of a subperiosteal implant (C) Custom-made milled subperiosteal implant. (D) Custom-made 3D printed subperiosteal implant.

DICOM images were imported into 3Diagnosys software (3DIEMME, Cantù, Italy) for nerve tracing and bone thresholding, and the resulting data were exported as an STL file. This file was then imported into Plastycad to develop a prosthetic plan with virtual abutments and to design a cutting guide for the mandible, which was subsequently 3D printed. Virtual grooves for the abutment sites were created on the STL model, and a frame for the subperiosteal implant was designed to ensure that screw holes avoided vital structures, such as the IAN. The final digital design was exported as an STL file for fabrication by CAD/CAM milling or 3D printing (Figures 1, 2).

3D printing (M-150T 3D printer, Guangzhou Riton Additive Technology Co., China) was carried out through additive manufacturing, utilizing selective laser melting to fuse titanium powder layer by layer with a high-powered laser. Post-processing included heat treatment and surface finishing (sandblasting/acid etching) to enhance biocompatibility and osseointegration. Milling was performed via precision machining of titanium blocks using high-precision CNC milling (3-axis, MILLSTAR, Taiwan) at low cutting speeds (20-60 m/minute) with carbide- or diamond-coated tools to avoid work hardening, employing water-soluble coolants for heat dissipation, along with adaptive toolpath strategies to ensure accuracy. After machining, implants underwent sandblasting and acid etching to improve osseointegration before cleaning and sterilization. 

Medical Titanium grade 4 was used either as a preformed block for milling or as a powder for additive manufacturing. The final manufactured frame thickness reached 1.5 to 1.2 mm, which measured around 1 mm after finishing and polishing. After fabrication, the implant was anodized (an electrochemical process that creates an oxide layer), cleaned with enzymatic solutions, subjected to ultrasonic cleaning to remove organic residues, packaged in double sterilization pouches, and sterilized in preparation for surgery. The sterilization protocol involved using a Class B autoclave vacuum cycle at 134 °C for 18 minutes. 

Before surgery, patients rinsed with 0.12% chlorhexidine mouthwash for one minute, followed by scrubbing of the intraoral and extraoral regions with povidone-iodine-soaked gauze, and were then draped with sterile surgical drapes. Procedures were performed under local anesthesia (4% articaine hydrochloride with 1:100,000 epinephrine; Artinepsia 4%, Spain) using inferior alveolar, lingual, and long buccal nerve blocks.

**Figure 3:**
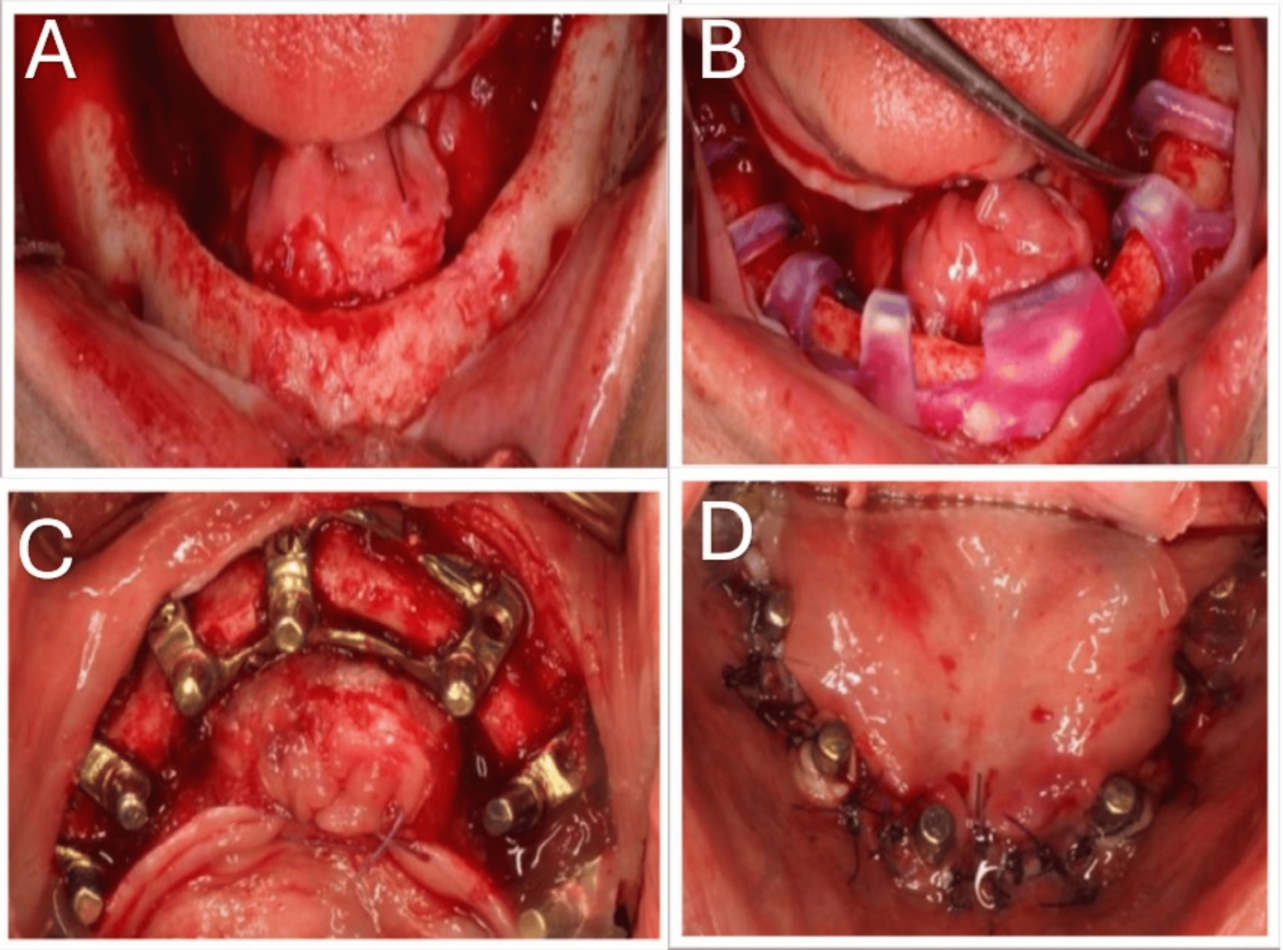
Surgical steps for the subperiosteal implant. (A) Mucoperiosteal flap incised and elevated. (B) Custom-made cutting guide in place. (C) Cortically fixed custom-made subperiosteal implant using 2.0 Mini screws. (D) Suturing of the flap around transmucosal abutments.

**Figure 4:**
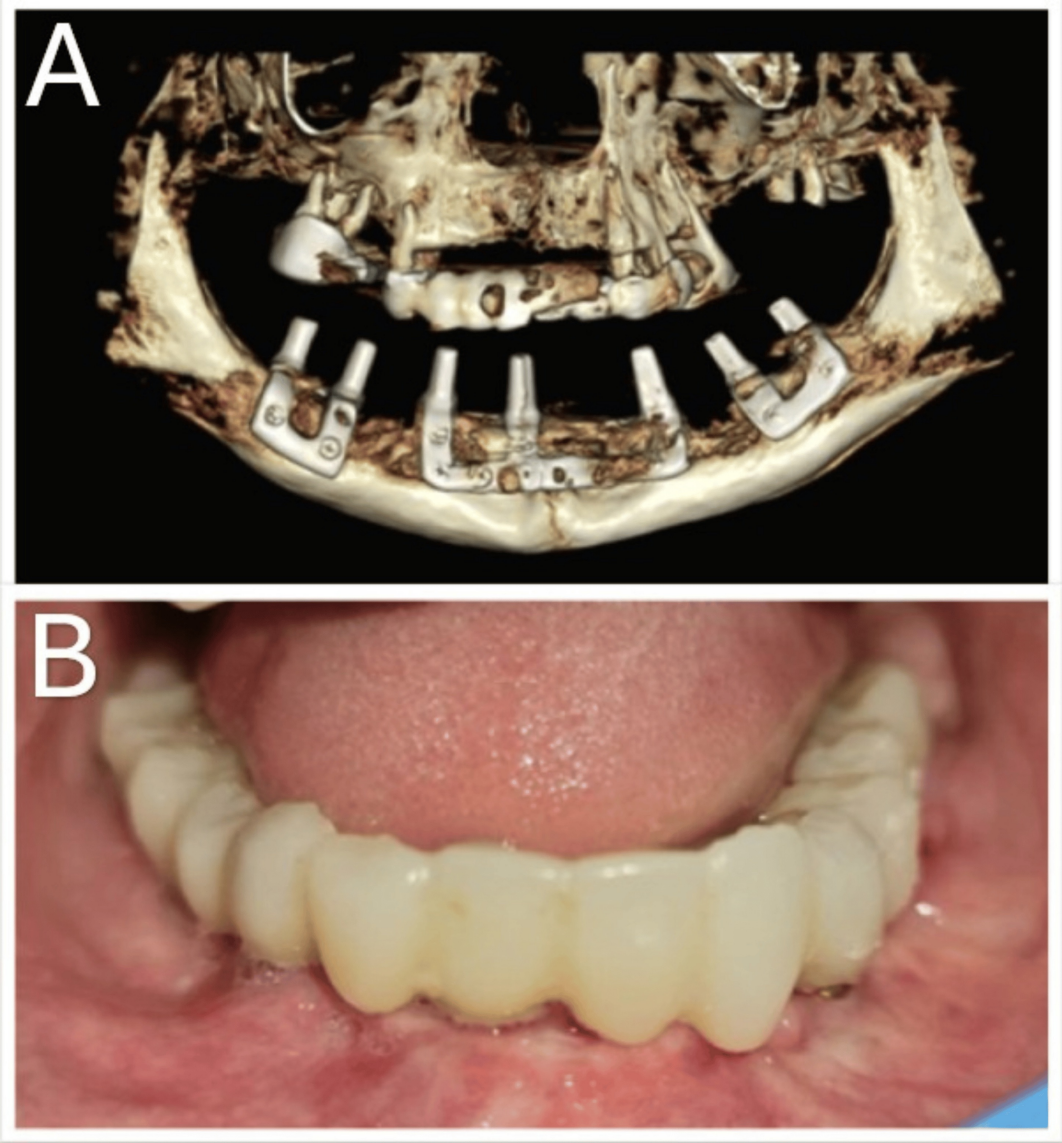
(A) Postoperative CBCT scan and (B) definitive cement-retained, resin-reinforced bridge after six months.

After achieving adequate anesthesia, a full-thickness mucoperiosteal flap was raised at the mid-crestal position using a single, precise incision made with a No. 15 scalpel blade. The flap was elevated with a periosteal elevator, and the custom cutting guide was positioned to ensure proper seating before osteotomies were performed with high-speed surgical burs. Once the osteotomies were completed, the implant seating (fabricated by either 3D printing or milling) was inspected for interference, and adjustments were made to allow passive seating. A 1 mm micro drill prepared the osteotomy bed for a 2 mm self-drilling screw, and each implant segment was secured with at least four mini screws on the buccal side and two on the lingual side. Finally, the flap was repositioned and sutured with simple interrupted stitches using 3/0 Vicryl thread. Postoperatively, patients received written care instructions and were prescribed antibiotics, analgesics, and anti-edema medications for at least five days (Figures 3, 4). 

Outcomes

The primary outcomes of this study were implant survival, bone resorption, and accuracy. The secondary outcomes included the duration of the intervention (in minutes) and soft tissue dehiscence (implant exposure). Implant survival was categorized into success and failure. All subperiosteal implants that functioned adequately at the 12-month follow-up without any complaints from the patient were considered successful and assigned a score of 1. Lost Implants were classified as failed and assigned a score of 0. Bone resorption and accuracy were measured through CBCT scans one week after surgery and again at 12 months postoperatively.

The procedure's accuracy was assessed in degrees. We measured the angular deviation between the abutments of the preoperatively planned STL design and those of the segmentation STL obtained from the immediate postoperative CBCT for each patient after superimposing both scans. 

Bone resorption involved two measurements taken using CBCT: the first was obtained immediately after surgery to assess the accuracy of crestal groove preparation by measuring the gap between the implant's fitting surface and the underlying bone. The second measurement, taken 12 months postoperatively, evaluated bone resorption stability by examining the same gap. Changes in bone stability between the 12-month and one-week postoperative CBCT were calculated to reflect bone resorption.

The duration of the intervention was measured from the start of local anesthesia administration until the flap was sutured. Soft tissue dehiscence was dichotomized into present or absent and measured at 3, 6, 9, and 12 months.

Sample size

The sample size calculation was based on previous studies [17-19]. Using a two-tailed 5% level of significance, 95% power, and an expected standard deviation (SD) of 0.1, the sample size for each group was calculated to be 10 patients.

Randomization and blinding

The allocation sequence was generated using a computer-generated random sequence by Random.org and a 1:1 allocation ratio. It was concealed using sequentially numbered opaque sealed envelopes containing folded paper with the type of PSI written on it. The allocation sequence generation and concealment were conducted by a researcher who was not involved in the surgical procedures or the outcome assessment. This study was a single-blinded trial with only the outcome assessor blinded since the operator and the patient were aware of the intervention due to its nature.

Statistical methods

Statistical analysis was performed using the Statistical Package for Social Sciences program (SPSS Inc., Chicago, IL). Numerical data were described as means and SDs. Categorical data were summarized as proportions. Continuous data were compared using an independent t-test. Categorical data were compared using the chi-square test. The level of significance was set at *P* < 0.05.

## Results

**Figure 5:**
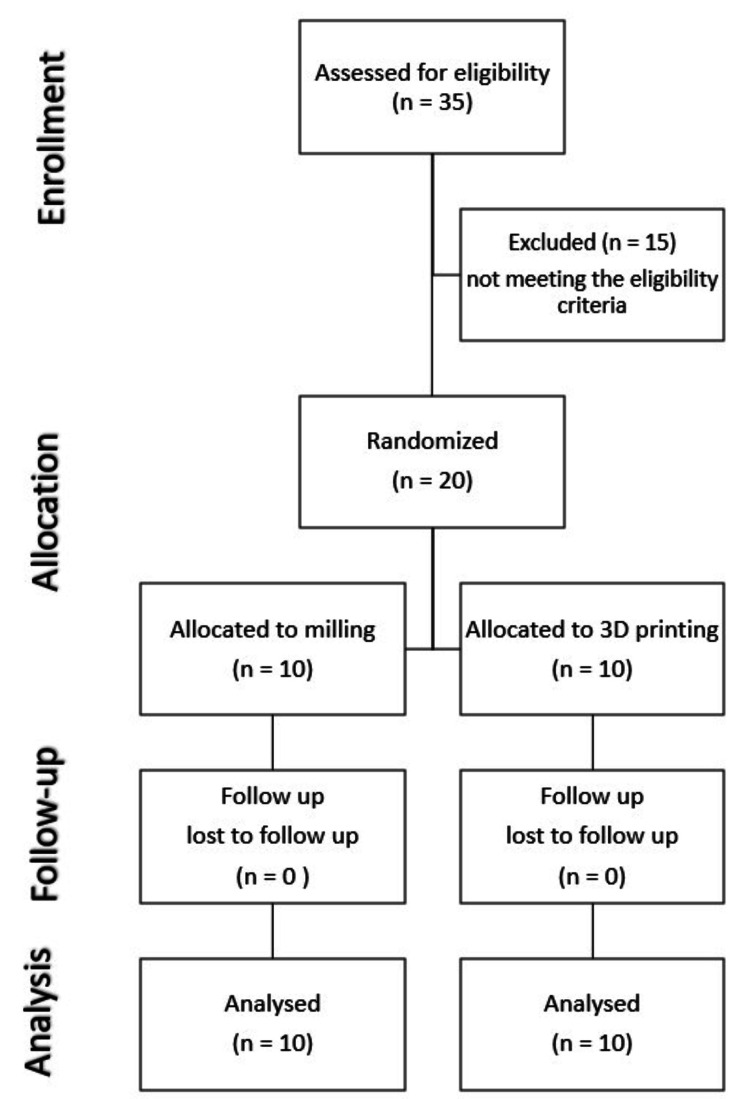
CONSORT flow diagram. CONSORT, Consolidated Standards of Reporting Trials

Twenty patients with severe atrophic edentulous mandibles were enrolled and randomized to receive PSI manufactured by milling or 3D printing. All patients completed the follow-up successfully (Figure 5). 

Regarding the implant survival rate, there was no statistically significant difference (*P *= 0.869) between the two groups after 12 months, with only one implant failing in the milling group. The accuracy of the PSI was measured by mesiodistal angular deviation and buccolingual angular deviation, with no statistically significant difference between the two groups regarding these parameters. Changes in bone resorption between the 12-month CBCT scan and the one-week postoperative CBCT scan showed no statistically significant difference between the two groups. Table 1 shows the two groups' mean and SD values for bone resorption and angular deviation. 

**Table 1:** Results for bone resorption and angular deviation in both groups. NS, nonsignificant; SD, standard deviation

Group	Mean	SD	t-value	*P*-value
Bone resorption (mm)				
Milling	0.1	0.317	1.47	0.0788 NS
3D Printing	0.04	0.0549		
Mesiodistal angular deviation (degree)				
Milling	2.86	1.89	1.119	0.1396 NS
3D Printing	2.01	1.22		
Buccolingual angular deviation (degree)				
Milling	4.38	6.17	0.834	0. 2082 NS
3D Printing	2.63	1.31		

As shown in Table 2, there were no statistically significant differences between the milling and 3D printing groups regarding the duration of the intervention and the soft tissue dehiscence at different time points.

**Table 2:** Results for the duration of the intervention and soft tissue dehiscence. NS, non-significant; SD, standard deviation

Group	Mean	SD	t-value	*P*-value
Duration of the intervention (minutes)				
Milling	104.6	9.48	0.896	0.198 NS
3D printing	99.2	9.58		
Soft tissue dehiscence (presence or absence)				
Timepoint (months)	Milling group (No/Yes)	3D printing (No/Yes)	Chi-square value	*P*-value
3	No: 9, Yes: 1	No: 10, Yes: 0	1.053	0.305 NS
6	No: 7, Yes: 3	No: 10, Yes: 0	3.529	0.060 NS
9	No: 7, Yes: 3	No: 10, Yes: 0	3.529	0.060 NS
12	No: 7, Yes: 3	No: 8, Yes: 2	0.202	0.653 NS

## Discussion

This study included 20 patients randomized into two groups. We selected patients with ridge heights less than 6 mm, focusing on those with significant anatomical limitations. Subperiosteal implants are ideal for cases where bone grafting or extensive ridge augmentation is not feasible, especially in older patients. Bone grafting carries risks such as infection, complications, costs, and longer treatment times, which are more significant in elderly patients with severe atrophy. Additionally, significant bone loss can prevent the use of short implants [20-22].

High-quality computed tomography and digital planning enable precise implant placement [23]. The implants' positioning depends on the dental arch's shape and the interocclusal space, ensuring proper function, aesthetics, and prosthetic fit. Consequently, a complete denture was fabricated for each patient preoperatively, and CBCT scans were performed with the denture in place [23,24]. This study used 3D-printed resin surgical guides to create pilot grooves in the crestal bone. These grooves enhance implant fit and accuracy, reduce surgical errors, alleviate soft tissue tension during closure, lower the risk of postoperative dehiscence, minimize micromotion, and evenly distribute occlusal forces, thereby reducing stress linked to bone resorption and implant failure [21,25].

Our study employed two manufacturing processes for PSI: CNC milling of titanium grade 4 for group A and 3D printing of titanium powder for group B. CNC milling was chosen for its high precision and smooth finish. At the same time, Direct Metal Laser Sintering (DMLS) was selected for its capability to produce complex, patient-specific designs with excellent mechanical properties. Both methods are preferred over traditional techniques for creating titanium bars for implant-supported hybrid denture frameworks [26]. All implants underwent anodization following fabrication to enhance biocompatibility and corrosion resistance [27].

In this study, an implant was deemed successful if it functioned properly without patient complaints at a one-year follow-up. The 3D printing group achieved a 100% survival rate (10/10 implants), while the milling group had a 90% survival rate (9/10 implants) after 12 months. The sole failure in the milling group was attributed to a patient's bruxism, resulting in an early loss due to mechanical stress. Statistical analysis (χ² = 0.027, *P* = 0.869) indicated no significant survival rate difference between the groups. The 3D printing results align with previous studies, including those by Mangano et al. and Mounir et al., reporting 100% survival rates [10,28]. Although the milling group's rate was slightly lower, it remains consistent with acceptable clinical outcomes, supported by findings from studies by Van de Borre et al. [29], El-Sawy and Hegazy [30], and Cerea and Dolcini [8], confirming high overall success for subperiosteal implants.

The accuracy of CAD/CAM-guided implant placement is reflected in the difference between planned and actual implant positions. These inaccuracies can occur at any stage: examination, planning, or execution [31]. In our study, the mean mesiodistal deviation was 2.86° ± 1.89° for the milled group and 2.01° ± 1.22° for the 3D-printed group. A t-test (t = 1.119, *P* = 0.1396) showed no statistically significant difference between these groups, suggesting clinically comparable results for mesiodistal angulation. For buccolingual deviation, the means were 4.38° ± 6.17° and 2.63° ± 1.31° for the milled and 3D-printed groups, respectively. Again, a t-test (t = 0.834, *P* = 0.2082) revealed no statistically significant difference.

Recent studies supported the use of surgical guides in implant surgery, with Al-Harbi and Sun [32] showing 88%-91% of implants had angular deviations under 7°. Shi et al.s' review [33] indicated mean distance deviations of <2 mm and angular deviations <8°, primarily <5°. Zhou et al. found significant differences in angular deviations between the maxilla and mandible, favoring flapless surgery with fixation screws [34]. Henprasert et al. noted no accuracy differences between additively and subtractively manufactured surgical guides [35]. Diaconu et al. found that virtual surgical planning in orthognathic surgery achieves acceptable deviations (<2 mm/4°), with patient-specific implants outperforming conventional methods [36].

Our research aligns with earlier studies, indicating minimal crestal bone resorption around maxillary subperiosteal implants, averaging 0.2 mm after one year, comparable to endosseous implants [37]. Other studies corroborate these findings, showing stable implant integration despite initial gaps caused by technical factors [38]. Furthermore, Van den Borre et al. reported minimal bone loss with biomechanically tuned 3D-printed implants [39], and a four-year follow-up demonstrated no significant changes in the gap between the implant and underlying bone at the abutments, highlighting the stability of the implant-bone interface [38].

The study found no significant difference in the mean duration of interventions between the milling group (*M* = 104.6, SD = 9.48) and the 3D printing group (*M* = 99.2, SD = 9.58). In contrast to the study by Korn et al., which reported intervention times of 135, 146, and 127 minutes [40], our shorter durations may reflect differences in patient demographics and surgical complexity [8]. However, Mangano et al.'s much shorter time (44.3 minutes) might indicate fewer complex cases or different procedures [10]. 

At the three-month follow-up, a single case of dehiscence was noted in the milling group and none in the 3D printing group. The 3D printing group had fewer instances of dehiscence at six and nine months (0 vs. 3 in the milling group), although this difference was not statistically significant (*P* = 0.060). This could be due to the insufficient keratinized mucosa around the per-mucosal extensions, which might be a critical factor in these cases.

At 12 months, the 3D printing group had two dehiscences while the milling group had three, again with no significant difference (*P* = 0.653). The favorable soft tissue integration likely contributed to minimizing dehiscence, which may be attributed to the anodized titanium surfaces, crestal grooves, and reduced hardware on the ridge [41].

Other studies showed varied dehiscence rates. Van den Borre et al. reported partial exposure in 26 out of 40 patients without significant functional or aesthetic issues [29]. Mounir et al. found wound dehiscence in one of five titanium group patients with subperiosteal implants [28]. Rinaldi et al. documented two cases of arm exposure among 15 patients, with one resolving spontaneously [25]. Dimitroulis et al. observed five cases of metal frame exposure among 21 patients, three requiring frame replacement due to perceived bulkiness [42]. Gasparini et al. noted minimal mucosal exposure in one of 18 patients without functional repercussions [43].

The limitations of this study include a small sample size, which could limit statistical power by increasing the risk of type II error, and a relatively short follow-up period of 12 months, which may constrain the generalizability and long-term evaluation of outcomes. The variability in surgical techniques and the distinctions between milling and 3D printing processes could have introduced some confounding variables as well as the variability in bruxism management since we included patients with broad eligibility criteria. Concurrently, the reliance on CBCT imaging presents potential inaccuracies in measurement attributable to artifacts and patient movement, which could affect the outcomes measurements. Furthermore, despite the advancements in digital planning and manufacturing, inherent challenges, such as soft tissue dehiscence, bone resorption, and mechanical complications persist, emphasizing the necessity for larger studies with prolonged follow-up to thoroughly assess the efficacy of patient-specific subperiosteal implants in the rehabilitation of atrophic mandibles.

## Conclusions

In conclusion, the comparative evaluation of milled and 3D-printed patient-specific subperiosteal implants for atrophic mandibular rehabilitation demonstrated similar results regarding implant survival, minimal bone resorption, and acceptable angular deviations, highlighting the precision of digital planning and execution. The study also indicated that, while the duration of interventions varied between the two fabrication methods, both techniques achieved comparable clinical outcomes with limited soft tissue dehiscence. These key findings emphasize the feasibility of customized subperiosteal implants as a promising alternative for managing severely atrophic mandibles, offering valuable insights into optimizing surgical protocols and enhancing patient care.
